# Robust, maintainable, emergency invasive mechanical ventilator

**DOI:** 10.5935/0103-507X.20220383-en

**Published:** 2022

**Authors:** Paulo J. R. Fonte, Alberto Martinho, Américo Pereira, Andreia Gomes, Ângela Neves, Antero Abrunhosa, António Bugalho, António Gabriel-Santos, António Grilo, Carlos Carmo, Elsa Maltez, João Agostinho do Nascimento, João Goes, João Martins, João Pedro Oliveira, Jorge Pimenta, José Paulo Santos, Luís C. Gil, Luís Lopes, Mário Pimenta, Olga Moreira, Orlando Cunha, Pedro Pinheiro de Sousa, Pedro Póvoa, Sandra Cavaco-Gonçalves, Susana Barroso, Telmo G. Santos

**Affiliations:** 1 ICNAS Pharma - Institute for Nuclear Sciences Applied to Health, Universidade de Coimbra - Coimbra, Portugal.; 2 ISEC - Coimbra Polytechnic - Coimbra, Portugal.; 3 LIP - Laboratory of Instrumentation and Experimental Particle Physics - Coimbra, Portugal.; 4 UNIDEMI - Department of Mechanical and Industrial Engineering, NOVA School of Science and Technology, Universidade NOVA de Lisboa - Caparica, Portugal.; 5 CIBIT - Coimbra Institute for Biomedical Imaging and Translational Research, Universidade de Coimbra - Coimbra, Portugal.; 6 Centro Hospitalar e Universitário de Coimbra, EPE, Universidade de Coimbra - Coimbra, Portugal.; 7 Escola Superior de Enfermagem de Coimbra - Coimbra, Portugal.; 8 CHRC - Comprehensive Health Research Centre, NOVA Medical School, Universidade NOVA de Lisboa - Lisboa, Portugal.; 9 Hospital CUF Tejo - Lisboa, Portugal.; 10 Magnamed - Comercialização de Produtos Médicos, Lda - Lisboa, Portugal.; 11 Médicos do Mundo - Lisboa, Portugal.; 12 Departamento de Engenharia Electrotécnica e de Computadores, Centro de Tecnologia e Sistemas, Faculdade de Ciências e Tecnologia, Universidade NOVA de Lisboa - Caparica, Portugal.; 13 Instituto Nacional de Investigação Agrária e Veterinária, I. P. - Oeiras, Portugal.; 14 Centro de Investigação Interdisciplinar em Sanidade Animal, Faculdade de Medicina Veterinária, Universidade de Lisboa - Lisboa, Portugal.; 15 Laboratory of Instrumentation, Biomedical Engineering and Radiation Physics, Department of Physics, Faculdade de Ciências e Tecnologia, Universidade NOVA de Lisboa - Caparica, Portugal.; 16 Haas F1 Team - Maranello, Italia.; 17 Polyvalent Intensive Care Unit, Hospital de São Francisco Xavier, Centro Hospitalar Lisboa Ocidental - Lisboa, Portugal.

**Keywords:** Ventilators, mechanical, Respiratory rate, Pulmonary ventilation, Continuous positive airway pressure, Oxygen, Volatile organic compounds, Gases, Animals

## Abstract

**Objective:**

To develop a simple, robust, safe and efficient invasive mechanical ventilator that can be used in remote areas of the world or war zones where the practical utility of more sophisticated equipment is limited by considerations of maintainability, availability of parts, transportation and/or cost.

**Methods:**

The device implements the pressure-controlled continuous mandatory ventilation mode, complemented by a simple assist-control mode. Continuous positive airway pressure is also possible. The consumption of compressed gases is minimized by avoiding a continuous flow of oxygen or air. Respiratory rates and inspiration/expiration time ratios are electronically determined, and an apnea/power loss alarm is provided.

**Results:**

The pressure profiles were measured for a range of conditions and found to be adjustable within a ± 2.5cmH_2_O error margin and stable well within this range over a 41-hour period. Respiratory cycle timing parameters were precise within a few percentage points over the same period. The device was tested for durability for an equivalent period of four months. Chemical and biological tests failed to identify any contamination of the gas by volatile organic compounds or microorganisms. A ventilation test on a large animal, in comparison with a well established ventilator, showed that the animal could be adequately ventilated over a period of 60 minutes, without any noticeable negative aftereffects during the subsequent 24-hour period.

**Conclusion:**

This ventilator design may be viable, after further animal tests and formal approval by the competent authorities, for clinical application in the abovementioned atypical circumstances.

## INTRODUCTION

The sudden worldwide onset of the coronavirus disease (COVID-19) pandemic declared in March 2020 by the World Health Organization (WHO) caused a global disruption in almost every area of the social, industrial and medical fields. As a consequence, a scarcity of invasive mechanical ventilators for respiratory support due to an increase in severe acute hypoxemic respiratory failure caused by severe acute respiratory syndrome coronavirus 2 (SARS CoV 2) was on the news all over the world. The scientific/technical community responded to this challenge with many designs of emergency ventilators to support the pandemic response in a multitude of working principles and technical implementations.^(1-10)^

One such effort,^(10)^ coauthored by many of the present authors, concentrated on safety and manufacturability in an environment of severely restrained international commerce and disruption of the industrial supply chains. Some of the technical options then adopted, during the first peak of the pandemic, are no longer relevant, as the industrial capacity has been restored and the medical equipment stocks have been reinforced. However, as a side consequence of this effort, some of the specific design choices born from a moment of great need may be useful in other contexts beyond the emergency response to COVID-19.^(11)^

It is clear that modern commercial ventilators generally provide a level of versatility, safety and ease of use that cannot be compared with most of the emergency models developed for COVID-19. Nevertheless, in remote areas of the world and in war zones, the practical utility of such sophisticated equipment is limited by considerations of maintainability, availability of parts, transportation and/or cost.

The ventilator design reported in this article implements a pressure controlled continuous mechanical ventilation (PC-CMV) mode, complemented by a simple assist control (PC-A/C) mode. Continuous positive airway pressure (CPAP) is also possible. Pressure-controlled ventilation is safe and well established in clinical practice, and is technically easy to implement, consistent with the objectives and needs.

## METHODS

The device is made from robust off the shelf industrial components, low tech machined parts and is controlled by simple hard wired feedforward electronics, without recourse to microcontrollers or computers. Although reducing the versatility and accuracy of the device, this choice was made to keep the technical complexity to a minimum, compatible with its intended use in atypical scenarios where access to standard equipment is restricted.

The pressure control elements are essentially passive for safety and reliability while providing a precise electronically determined range of respiratory rates and inspiration/expiration time ratios (I/E). Apnea and power loss alarms are also incorporated.

In addition to the intrinsic robustness, part sourcing and maintenance can be done at a relatively low level of technical expertise, rendering it suitable for use in remote locations with little access to sophisticated technical support. To reduce dependencies, no consumables are needed except those in the patient respiratory circuit. The availability of compressed gases, usually logistic distress, is mitigated by avoiding a continuous flow of oxygen or air so that all of the gas used is actually inhaled by the patient.

**Figure 1:**
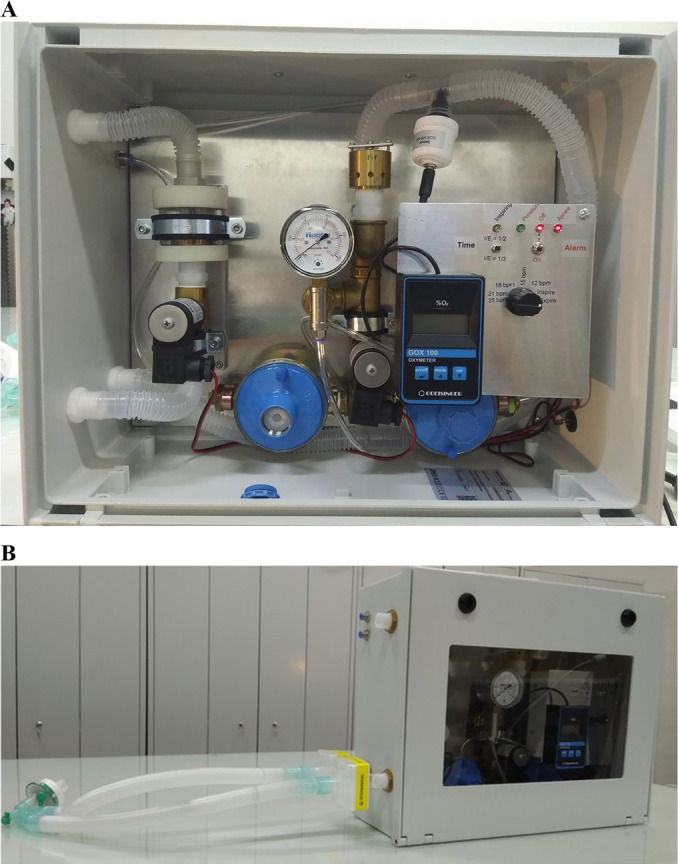
Practical implementation of the ventilator. (A) Internal view showing the command panel and the main components. (B) External view with the attached standard double-limb respiratory circuit and, on top, the exhaust outlet and air and oxygen intakes (blue connectors).

The practical implementation of the ventilator is depicted in figure 1. For brevity, the technical description of its constitution and working principle is provided in Appendix 1S (Supplementary material).

PIP - peak inspiratory pressure; PEEP - positive end-expiratory pressure; bpm - breaths per minute; I/E -inspiration/expiration time ratio; FiO_2_ - fraction of inspired oxygen.

To ascertain the safety and efficacy of the ventilator, we performed physical, chemical and bacteriological tests, supplemented with animal nonclinical studies, by the methods described below.

### Physical tests

As this ventilator implements only the pressure controlled ventilation mode, the main physical characteristic to be registered is the pressure profile applied to the endotracheal tube as a function of time, which was measured at the entrance of the “Y” piece (junction of the inspiratory and expiratory branches).

The other main physical parameter, the oxygen fraction present in the gas, was measured in the inspiratory branch.

The pressure was measured by an electronic manometer Honeywell HSCDRRN001BDSA3 (± 1% accuracy), and the oxygen fraction was measured by visual observation of a sensor Greisinger GOX 100T. The ventilator was coupled to a Siemens lung model Test Lung 190, with an estimated compliance of 30mL/cmH_2_O. For all tests, the heat and moisture exchanger *filter* and the bacterial filters on each disposable airway circuit were inserted, and the tubing was fully extended.

The configurable parameters were varied to cover the extremes of the parametric space, as systematized in table 1. The normal pressure ranges lie between 10 and 30cmH_2_O for peak inspiratory pressure (PIP) and between 0 and 20cmH_2_O for positive end-expiratory pressure (PEEP). In all cases, both pressures were adjusted by acting on the corresponding adjustment knobs and monitoring the values on the mechanical manometer and oxygen sensor. Higher PIP pressures can be achieved by replacement of the internal spring of the pressure regulator by a stiffer one at the expense of a brisker adjustment at the lower PIP range. The safety valve can be adjusted accordingly without further modification.

**Table 1:** List of the pressure profile measurements, covering the limits of the space of configurable parameters

PIP (cmH_2_O)	PEEP (cmH_2_O)	Respiratory rate (bpm)	I/E	FiO_2_
10	0			
30	0	12, 18 or 25	1/2 or 1/3	50% ± 5% or 100%
30	20			

bpm - breaths per minute; I/E -inspiration/expiration time ratio.

The respiratory rate lies between 12 and 25 cycles per minute, and I/E can be selected for the values 1/2 and 1/3. These quantities are rigidly determined by electronic circuits and were measured from oscilograms of the command signals of the e-valves, from which the timing information is derived by mathematical analysis.

For all measurements, the working gases were oxygen at a pressure of 4 bar and air at the same pressure.

The fraction of inspired oxygen (FiO_2_) can be adjusted between 21% and 100% with a typical accuracy of ± 5%. For these tests, we covered FiO_2_ = 100% or FiO_2_ = 50% ± 5 %.

For the stability assessment, the pressure profiles taken at a respiratory rate of 25bpm were recorded for 15 s every 10 minutes over a period of 41 hours, and the evolution over time for the PIP, PEEP and respiration frequency was deduced mathematically by waveform analysis.

The operation of the safety valve, limiting the airway pressure to 45cmH_2_O, was performed in CPAP mode at 30cmH_2_O. An overpressure in the airways was created by the application of an external weight to the test “lung” followed by its removal and repressurization while observing the pressure profile at the Y junction.

In the assisted ventilation mode, any inspiration effort that brings the airway pressure lower than -2cmH_2_O triggers a new respiratory cycle. In absence of such efforts, the system defaults to a 10bpm respiratory rate. A short test (approx. one minute) was made on a conscious human subject (one of the authors) at 50% FiO_2_, PIP = 15cmH_2_O and PEEP = 5cmH_2_O. The pneumatic coupling of the ventilator to the subject’s respiratory tract was made via a self-held noninvasive facial mask.

### Chemical and bacteriological tests

Prior to both the chemical and biological tests, the inspiratory branch was ultrasonically washed in deionized water plus neutral detergent, thoroughly rinsed in deionized water and dried in an oven at 60ºC for 12 hours.

### Volatile organic compounds

The quality of the oxygen/air mixture after passing though the ventilator was determined by gas chromatography-mass spectrometry (GC-MS) to look for volatile organic compounds that could arise from the materials used in its construction.

A pressurized oxygen/air mixture was passed through the equipment and fed to a sampling tube filled with sorbent (Supelco ORBO 43) for 1 hour. During this time, the inspiration valve was operated at a frequency of 120 cycles/minute.

After sampling, the tube was closed, kept at 3ºC to 5ºC, and protected from light until the GC MS analysis. The sample was extracted from the sorbent with toluene and analyzed. A baseline sample of the oxygen/air fed to the ventilator was also similarly collected and analyzed for comparison.

Toluene was obtained from Fisher Chemical (UK) and used without further purification.

Gas chromatography‒mass spectrometry analysis was performed in a GC-MS QP-2010 Plus from Shimadzu. Injections were performed using an AOC-5000 Auto Injector and a Supelco SLB 5ms fused silica capillary column, 60m × 0.25mm ID, fused silica capillary, 0.25µm. Data acquisition and analysis were performed with LabSolutions - GCM Solution version 2.50 software SU3.

Chromatographic conditions: injector temperature: 200ºC; detector temperature: 250ºC; interface temperature: 290ºC; oven temperature programme: 130°C to 290°C at 4ºC for min (hold 20 minutes at the end); transporter gas: He; linear velocity: 35cm/sec; injection volume: 1µL; split ratio: 1.0.

### Bacteria and fungi

Under the same conditions as for the chemical tests, a mixture of oxygen/air passing through the ventilator was bubbled for five minutes in water for injectables obtained from B. Braun Medical (Portugal) and used without further purification.

Samples were incubated in YEA (yeast extract agar) and Rose-Bengal with chloramphenicol agar base (according to ISO 6222) at 22ºC for 2 days and 36ºC for 3 days for the detection of microorganisms and in MEA (malt extract agar) at 25ºC for 5 days for fungi (ISO 21527).

### Animal studies

To address the safety and efficacy of the ventilator, *in vivo* studies were performed in a porcine model, owing to its size and similarity with the human lungs.

This study was performed at *Estação Zootécnica Nacional* of the Portuguese *Instituto Nacional de Investigação Agrária e Veterinária* (INIAV I.P.), the 17^th^ November 2020. The study was approved by the Authority Responsible for Animal Welfare of INIAV I.P. (ORBEA-INIAV), and authorization for animal experimentation (nº 0421/2020) was obtained from the General Directorate of Food and Veterinary (DGAV). All handling and care followed the European directive 2010/63/EU on the protection of animals used for scientific purposes and Good Laboratory Practices (GLP).

After a food (12 hours) and water (3 hours) fasting period, a 5-month-old pig (*Sus scrofa domesticus*), weighing 52kg, received midazolam (0.5mg/kg intramuscular) as a pre anesthetic medication followed fifteen minutes later by anesthesia induction with intramuscular administration of dexmedetomidine (0.01mg/kg), morphine (0.2mg/kg) and ketamine (15mg/kg).

Ten minutes later, after being placed on a warmed surgical bed, an intravenous catheter was placed in the marginal vein of the ear, and the animal was further anesthetized with propofol (3.5mg/kg). This access was kept for continuous administration of propofol as anesthetic maintenance.

After the loss of the swallowing reflex, the animal was intubated with an endotracheal tube (nº 6.5) and connected via a double limb respiratory circuit to a reference Manley type^(12)^ mechanical ventilator manufactured by the BLEASE company, which is a volume-controlled, pressure-limited, well-known classical ventilator. This ventilator was retrofitted with a supplementary PEEP valve similar to V4 (Appendix 1S - Supplementary material).

Indeed, the test involved three ventilators: Ventilator 1, the BLEASE; Ventilator 2, the ventilator object of this paper; Ventilator 3, another minimalist and low-cost ventilator not part of this report.

Permanent access to arterial blood was assured via an arterial line inserted in the femoral artery. Arterial blood gases (ABG) were measured regularly by an Abbott i-STAT CG8+ blood gas analyzer yielding the arterial partial pressure of oxygen (PaO_2_), arterial partial pressure of carbon dioxide (PaCO_2_), bicarbonate concentration (HCO_3_), arterial oxygen saturation (SaO_2_) and acid-base balance (pH).

Peripheral oximetry (SpO_2_) and heart rate (HR) were continuously monitored by a pulse oximeter (BCI International Model 71200) applied to the tongue of the animal, and rectal temperature was measured with an electronic thermometer.

Supplementing the instruments on the ventilators, for the purpose of monitoring the experiment, we had an external electronic manometer, spirometer and line oximeters inserted in the inspiratory line measuring PIP, PEEP, tidal volume (V_T_), FiO_2_, respiratory rate and I/E. However, all ventilator settings adjusted during the test were applied using only the information from the instrument’s own dials.

## RESULTS

### Physical tests

#### Timing accuracy

The electronically measured values of the respiratory rate and the I/E ratio are reported in table 2 and are compared with the nominal values, showing relative deviations of only a few percent. These deviations are within the range spawned by the finite precision of the values of the electronic components.

**Table 2:** Measured values of respiratory rate (bpm) and ratio of inspiration time to expiration time (I/E)

Respiratory rate (bpm)			I/E (%)					
**Nominal**	**Measured**	**% Deviation**	**Nominal**	**Measured**	**% Deviation**	**Nominal**	**Measured**	**% Deviation**
25	24.7	-1.2	75	78	-4.00	66.6	63	5.41
21	20.7	-1.4	75	77	-2.67	66.6	63	5.41
18	18.2	1.1	75	77	-2.67	66.6	63	5.41
15	15.0	0.0	75	77	-2.67	66.6	63	5.41
12	12.3	2.5	75	77	-2.67	66.6	63	5.41

#### Pressure profiles

**Figure 2:**
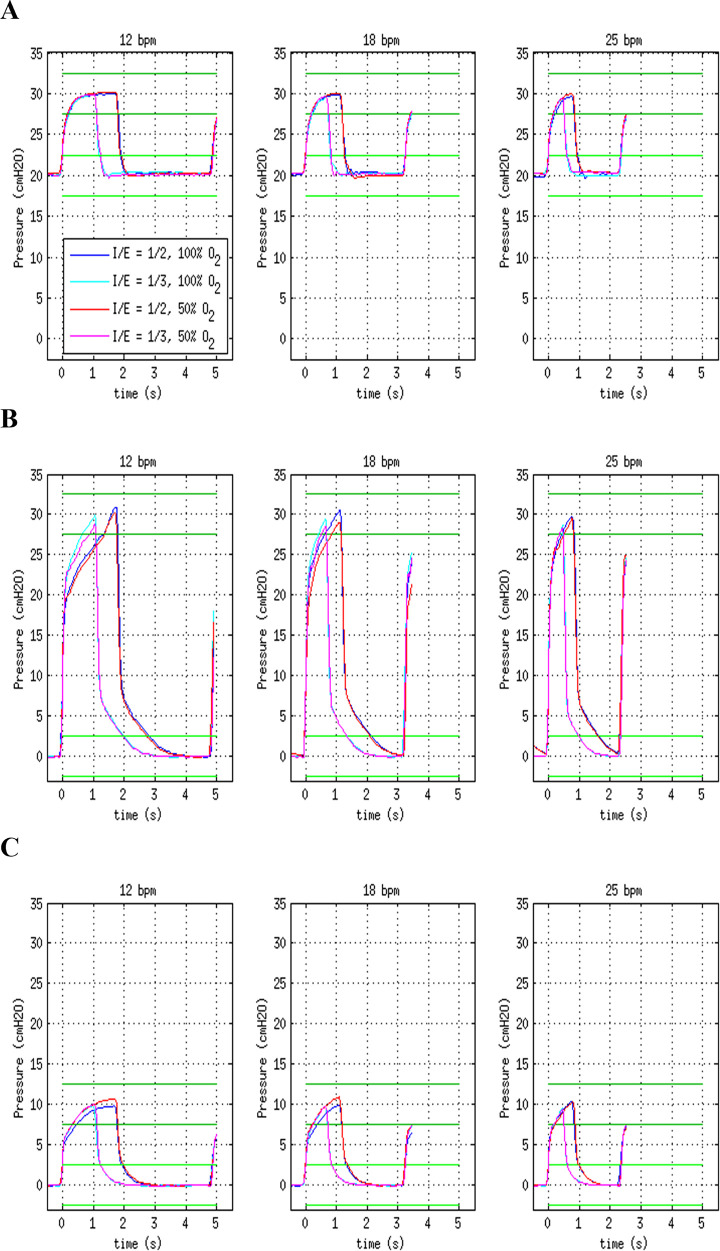
Instantaneous pressure at the Y junction, represented as a function of time after the beginning of the inspiratory cycle, for respiratory rates of 12, 18 and 25 bpm, 50% ± 5% oxygen concentration (red curves) or 100% oxygen (blue curves) and I/E ratio of 1/2 (darker curves) or 1/3 (lighter curves). (A) PIP = 30cmH_2_O, PEEP = 30cmH_2_O; (B) PIP = 30cmH_2_O, PEEP = 0cmH_2_O; (C) PIP = 10cmH_2_O, PEEP = 0cmH_2_O. The regions between the green horizontal lines define the acceptable accuracy range (see text) for PIP (upper, darker green lines) and PEEP (lower, lighter green lines).

The instantaneous pressure at the Y junction as a function of time is shown in figure 2, covering the parameter combinations listed in table 1. Additionally, the acceptable ranges of PIP and PEEP for each case are also marked. These were defined as ± 2.5cmH_2_O, based on the requirement in the Medicines and Healthcare Products Regulatory Agency (MHRA) specifications^(13)^ that the adjustment of these pressures should have a maximum granularity of 5cmH_2_O, indicating that smaller steps are clinically not meaningful.

The pressure profiles generally feature a steep initial step, up to at least 50% of the pressure impulse, followed by a ramp until the set PIP is reached. For the slower respiratory rates, a pressure plateau can be reached, approximating the desirable “rectangular” profile. This behavior is determined by the characteristics of the pressure regulators, which are limited in maximum flow.

For all cases, the attained PIP and PEEP fell within the acceptable ranges, and the variation in the oxygen concentration had little effect on the pressure profiles.

#### Safety relief valve

**Figure 3:**
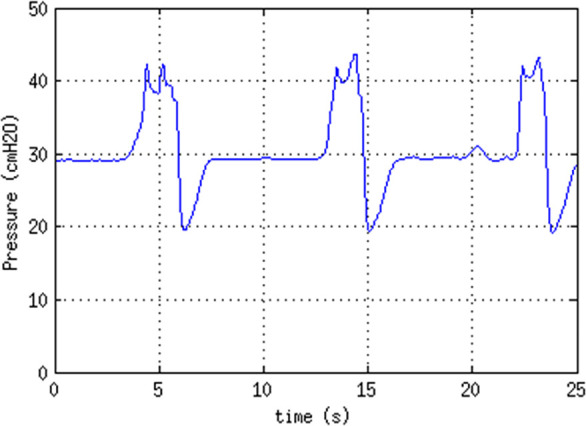
Action of the safety valve. Pressure in the Y-junction as a function of time, obtained in CPAP mode while repeatedly completely emptying the test lung by applying an external weight, followed by release and repressurization at PIP = 30cmH_2_O, evidencing the effective operation of the safety valve in maintaining a maximum PIP of 45cmH_2_O.

Evidence of the effective operation of the safety valve in maintaining a maximum PIP of 45cmH_2_O is shown in figure 3.

#### Assisted ventilation

**Figure 4:**
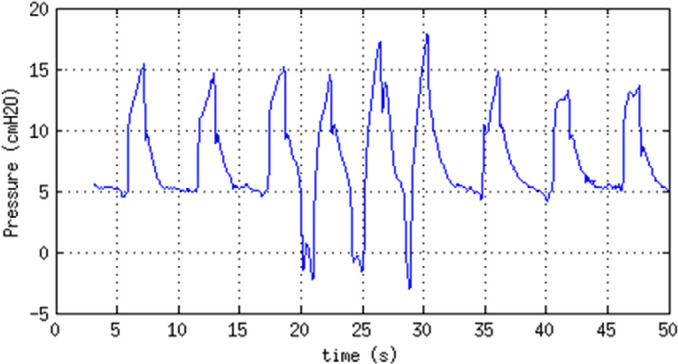
Demonstration of the assisted ventilation mode. Superimposed on a default respiration rate of 10 bpm, any inspiration effort that brings the airway pressure lower than -2cmH_2_O triggers a new cycle. Three such events are visible between 20s and 30s.

The operation of the assisted ventilation mode is demonstrated in figure 4.

#### Stability

**Figure 5:**
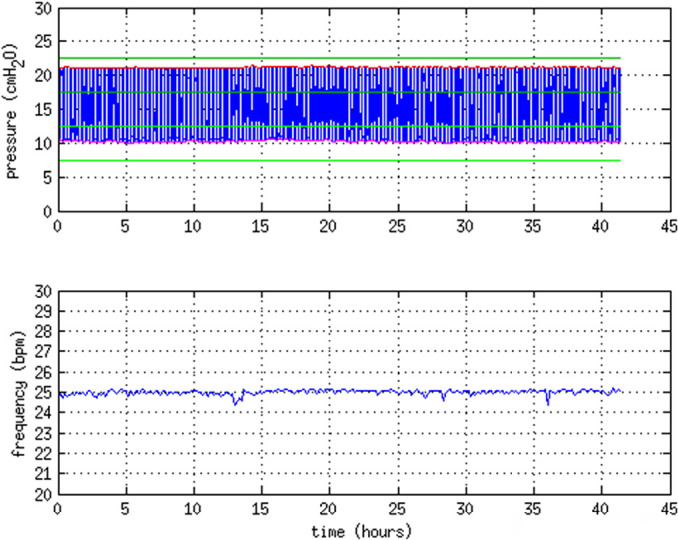
Stability test. Pressure curves recorded for 15s every 10 minutes over a period of 41 h. Upper panel - cumulative data, highlighting the maximum (PIP - red curve) and minimum (PEEP - pink curve) pressure values in each of the 15s recording periods. The acceptable PIP and PEEP accuracy ranges as defined in figure 2 are also displayed. Lower panel - the respiratory rate measured in each of the 15s recording periods.

The pressure profiles taken over a period of 41 hours are shown in figure 5. The stability of the pressures over this period was well within the ± 2.5cmH_2_O acceptable error range for PIP and PEEP. The respiratory rate was stable within 2% of the nominal value.

#### Durability

To derive a lower limit of the durability of the system in terms of the number of pressure cycles before failure, the system was operated at a very high respiratory rate for an extended period of time while pressurized at 4 bar of pure oxygen.

At the time of writing, the system had been operated for 28 days at a respiratory rate of 120 bpm without failure or degradation of characteristics, corresponding to four months of continuous operation at a normal respiratory rate of 25 bpm.

### Chemical and bacteriological tests

#### Volatile organic compounds

**Figure 6:**
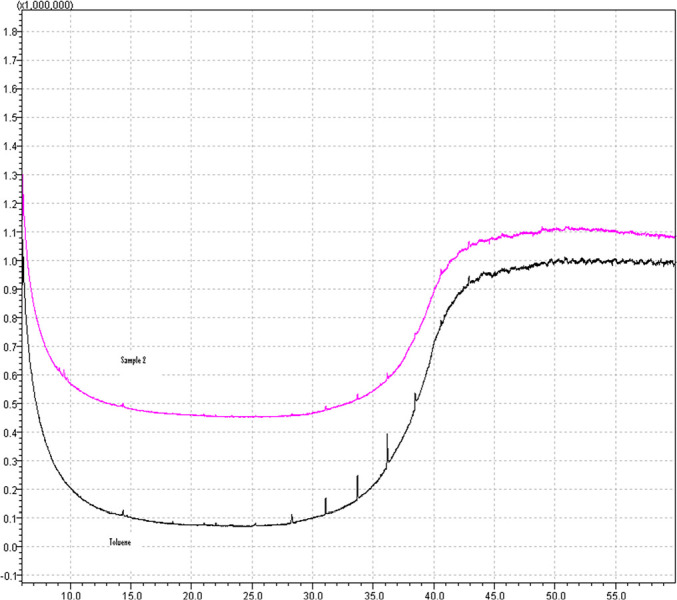
Volatile organic compounds. GC-MS spectrum from the gas mixture fed to the device (black curve) and the gas mixture that passed through (pink curve). Horizontal scale: retention time (minutes). Vertical scale: total ion current (arbitrary units).

The GC‒MS analysis of both the gas mixture fed to the device (baseline) and of the gas mixture that passed through (sample) is presented in figure 6, corresponding to a mass scan m/z between 30 and 300amu.

It can be seen that the baseline actually shows more traces of extraneous compounds than the sample. This is attributed to residual compounds introduced from the gas distribution system, as the baseline was collected before the sample. Overall, no extraneous compounds were introduced by the device.

#### Bacteria and fungi

All tests for bacteria and fungi were negative.

#### Animal studies

**Figure 7:**
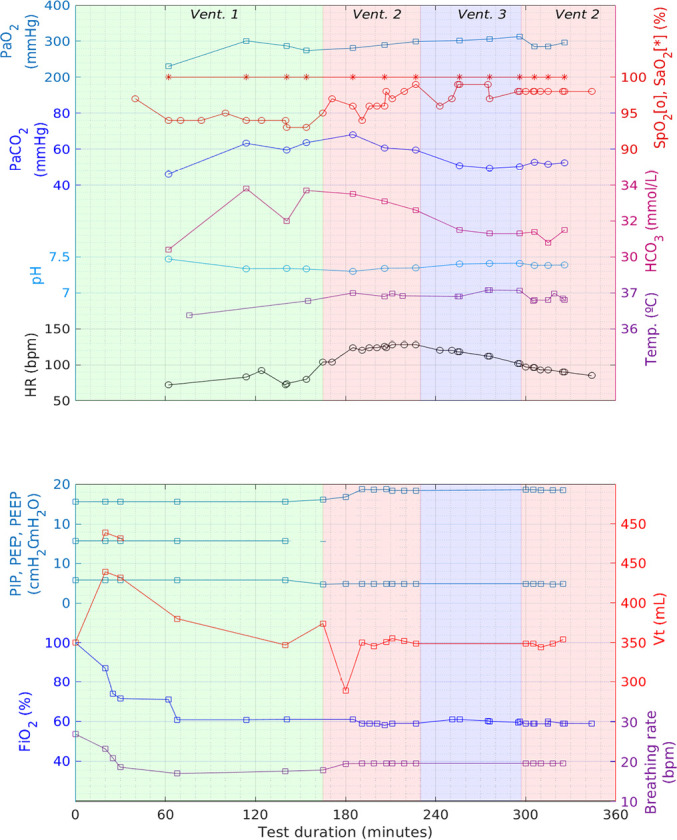
Main quantities of interest as a function of the time elapsed from the start of ventilation. The upper panel corresponds to the physiological parameters of the animal, and the lower panel corresponds to the physical parameters of the ventilators. See the text for the abbreviations. The lines are meant only to guide the eye. HR - heart rate; PaCO_2_ - partial pressure of carbon dioxide; PaO_2_ - partial pressure of oxygen; HCO_3_ - bicarbonate; SpO_2_ - peripheral oxygen saturation; SatO_2_ - oxygen saturation; FiO_2_ - fraction of inspired oxygen; PIP - peak inspiratory pressure; PEEP - positive end-expiratory pressure; V_T_ - tidal volume.

The main quantities of interest are plotted in figure 7 as a function of the time elapsed from the start of ventilation, corresponding to the upper panel to the physiological parameters of the animal and the lower panel to the physical parameters of the ventilators.

**Figure 8:**
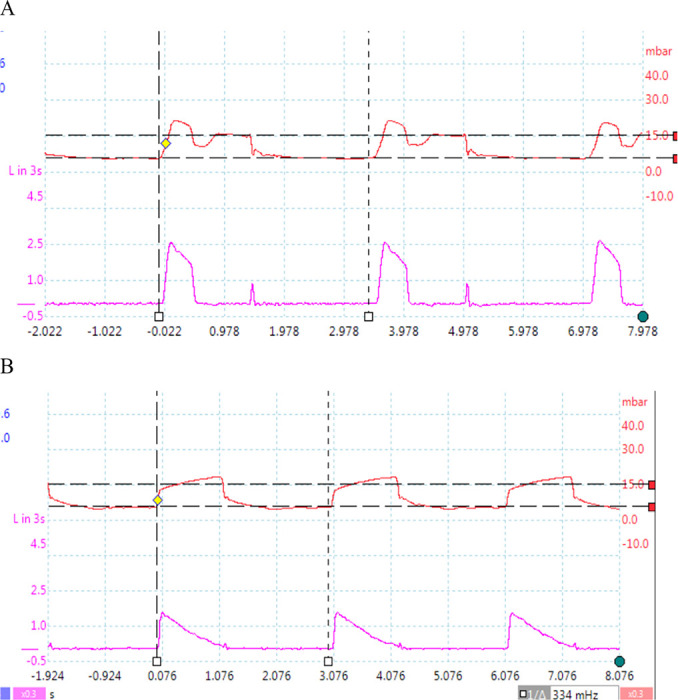
Example inspiratory pressure and flow curves. Upper panel: Ventilator 1 at minute 140. Lower panel: Ventilator 2 at minute 190. Note the markedly different curve shapes, indicative of the different controlled variables in each ventilator (volume *versus* pressure).

Representative examples of the instantaneous inspiratory pressure and flow are shown in figure 8.

The V_T_ was initially set at 350mL, in accordance with the accepted rate for this kind of animal (actually the same as for adult humans, 6 to 8mL/kg). The FiO_2_ was initially set at 100%, and PEEP was set at 6mbar. The V_T_ setting resulted in a PIP of approximately 16cmH_2_O (Figure 8).

The insertion of the arterial line took almost two hours, during which the oxygen saturation of the animal was monitored by a pulse oximeter. Some adjustment was necessary during this period, namely, a reduction of FiO_2_ and respiratory rate, as the animal appeared to be hyperventilated and suffering from hyperoxia.

Regular ABG was started after 114 minutes (Figure 7) and was maintained until a steady state in PaO_2_ was reached for a period of 40 minutes (3 ABG tests). At this point Ventilator 1 was replaced by Ventilator 2, which was set for approximately the same physical parameters: PIP, PEEP, respiratory rate and FiO_2_, at an I/E of 1/2.

As Ventilator 2 is a pressure-controlled ventilator, V_T_ becomes a secondary parameter determined by the physiology of the animal, causing the pressure and air flow profiles to differ markedly from Ventilator 1 (which is volume-controlled), as shown in figure 8. As the resulting V_T_ was somewhat decreased to 280mL, the nominal value of 350mL was restored by increasing PIP from 16 to 19cmH_2_0.

These conditions were maintained for a period of 36 minutes, during which three ABG procedures were performed, indicating a stable PaO_2_ and modest variations in the other blood gasometric parameters, demonstrating adequate ventilation conditions.

Between minutes 230 and 246, Ventilator 3 replaced Ventilator 2. During this period, the slightly upward trend in PaO_2_ and downward trend of PaCO_2_ and HCO_3_ continued, but the HR increase observed during the previous test was reversed.

Trying to ascertain whether this HR variation, although slow, was due to some characteristic of Ventilator 2 or it was caused by external conditions that happened to coincide in time with the test of Ventilator 2, after 300 minutes, Ventilator 2 was again used for another series of three ABGs spanning almost 30 minutes. During this period, the ABG quantities remained essentially stable, but the downward trend in HR continued, excluding any direct influence of Ventilator 2 on the initial increase in HR.

Afterward, the animal fully recovered from the anesthesia and was kept under observation for 24 hours. Its general state was normal, showing no signs or symptoms that could indicate any adverse event or adverse reaction to the intervention previously performed.

## DISCUSSION

The respiratory rates, with fixed values of 12, 15, 18, 21 or 25bpm, were electronically measured and found to be within 2.6% of the nominal values. The inspiration/expiration time ratio, with nominal values of 1/2 and 1/3, were likewise measured to lie within 5.4% of the nominal values. Over a 41-hour period, the respiratory rate did not deviate more than 2% from the nominal value.

The guidelines^(13)^ specify a range from 10 to 30 bpm in increments of 2 bpm, which corresponds to an accuracy of 2/30 (6.7%) in the most stringent case and implies that this is sufficient for the purpose. Although our device provides a slightly smaller range and wider steps (but it easily could be improved with just a modest addition of complexity), the accuracy remains better than shown in this figure.

The pressure profiles were measured as a function of time for several combinations of PIP and PEEP, covering the operational range of the device. In all cases, the required pressures, adjusted via the device’s own mechanical manometer, were attained within a ± 2.5cmH_2_O error margin when compared with an electronic manometer, within the limits defined by the “MHRA specifications”.^(13)^ The stability of PIP and PEEP was tested over a 41 h period, remaining well within this range.

The action of the mechanical overpressure safety valve required in the “MHRA specifications”^(13)^ was demonstrated. Although the set value was 40cmH_2_O, it could as well be set to the 80cmH_2_O mentioned in the same guidelines.

The operation of the optional pressure-controlled assisted ventilation mode (PC-A/C) was tested on a human subject and found to be effective.

At the time of writing, the prototype had been operated in pure oxygen for a number of cycles corresponding to four months of continuous normal operation at the maximum respiratory rate without any malfunction or degradation of characteristics. Although this is a significant time span for an emergency device, it may be necessary in the future to lengthen this study.

Chemical and biological tests were performed on the inspiratory branch to identify the introduction of volatile organic compounds or microorganism contamination of the gas. Both tests were negative, indicating that, although we used off-the-shelf industrial components, after adequate cleansing, these components do not present any obvious danger to human health.

A ventilation test on a large healthy animal, in comparison with a well established ventilator, showed that the animal could be adequately ventilated over a period of 60 minutes, as verified by ABG. The animal was observed for a subsequent 24-hour period without any noticeable negative aftereffects, establishing the *in vivo* safety and efficacy of the ventilator. Further work should include a similar test in a diseased animal model.

## CONCLUSION

We propose a simple, robust, safe and efficient invasive mechanical ventilator designed for use in remote areas of the world or war zones where the practical utility of more sophisticated equipment is limited by the considerations of maintainability, availability of parts, transportation and/or cost.

Physical, chemical, bacteriological and *in vivo* tests were performed on the prototype for instrumental verification of its physical characteristics and chemical/biological safety, as well as its safety and efficacy on a healthy large animal, with fully positive results. Further work should include a similar test in a diseased animal model.

Therefore, we conclude that this ventilator design may be viable, after further animal tests and formal approval by the competent authorities, for clinical application in the abovementioned atypical circumstances.
