# Supplementary material for: Robust, maintainable, emergency invasive mechanical ventilator
Source: Rev Bras Ter Intensiva. 2022 Jul-Sep;34(3):351–9. doi: 10.5935/0103-507X.20220383-en (PMC9749093; doi:10.5935/0103-507X.20220383-en)
Supplement: Supplementary file 1 [file rbti-34-03-0351-suppl01.pdf]

Robust, maintainable, emergency invasive mechanical ventilator

Ventilador mecânico invasivo de emergência resistente e de fácil manutenção

Paulo J. R. Fonte[1,2,3], Alberto Martinho[4], Américo Pereira[3], Andreia Gomes[5,6,7], Ângela Neves[1], Antero Abrunhosa[1,5], António Bugalho[8,9], António Gabriel-Santos[4], António Grilo[4], Carlos Carmo[10], Elsa Maltez[1], João Agostinho do Nascimento[11], João Goes[12], João Martins[12], João Pedro Oliveira[12], Jorge Pimenta[13,14], José Paulo Santos[15], Luís C. Gil[4], Luís Lopes[3], Mário Pimenta[3], Olga Moreira[13,14], Orlando Cunha[3], Pedro Pinheiro de Sousa[16], Pedro Póvoa[8,17], Sandra Cavaco-Gonçalves[13], Susana Barroso[1], Telmo G. Santos[4]

Appendix 1S - Technical description of the ventilator

Pneumatics

The schematic representation of the pneumatic circuit of the ventilator is shown in figure 1S. And its practical implementation is depicted in figure 2S, with identification of the main components and of the respiratory circuit.

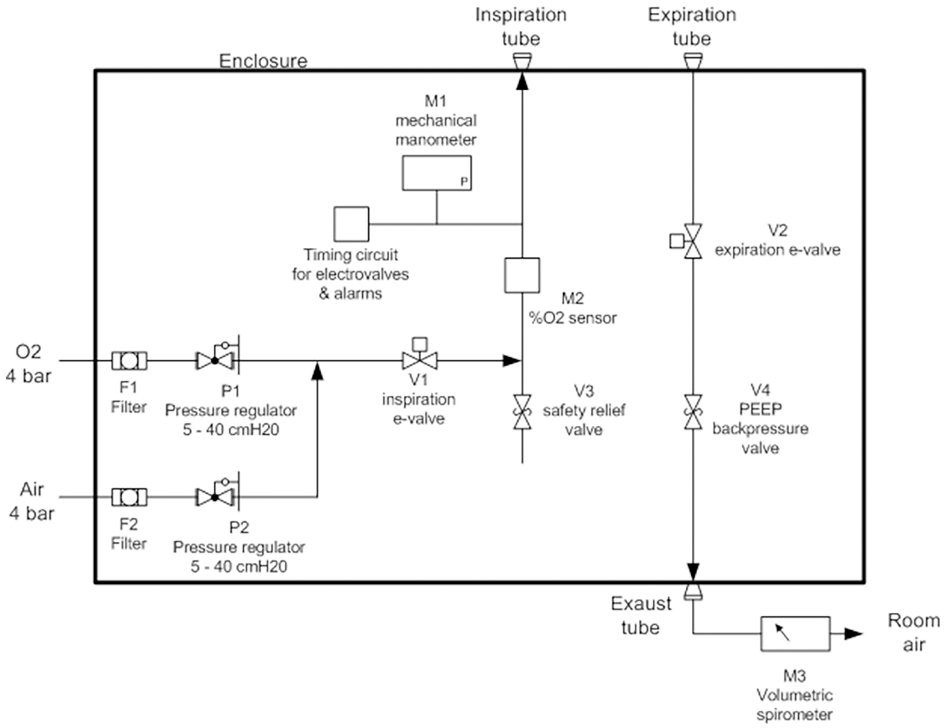

Figure 1S - Schematic representation of the pneumatic circuit.

The supply of air and oxygen (nominally at a pressure of 4bar, but acceptable in the range 1 to 10bar) enters from the connectors on the left and connects via dust filters[1] to the pressure regulators P1 and P2.

These are industrial[2] adjustable regulators that have an output range of 5 to 40cmH_2O, allowing the peak inspiratory pressure (PIP) to be set by the turn of a knob while observing the mechanical manometer M1. These pressure regulators benefit from more than 70 years of industrial development, are in widespread use for all types of gas installations and are quite safe (e.g. the regulators used for SCUBA diving are based on a similar principle and used since 1945). For instance, as verified by our own test, even a large rupture of the internal membrane will not produce an overpressure at the output.

The fraction of inspired oxygen (FiO_2) can be set by gently adjusting the relative pressure of both regulators while observing the oximeter M2 and the manometer M1. Once set, this value is quite stable.

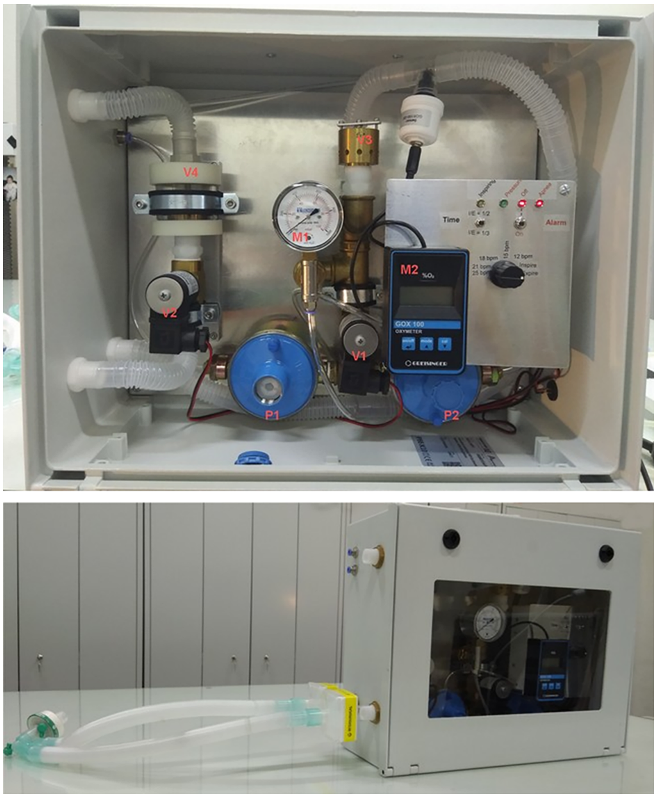

Figure 2S - Practical implementation of the ventilator.

Upper panel: internal view, with identification of the main components. Lower panel: external view with attached standard double-limb respiratory circuit and, on top, exhaust outlet (22mm Ø) and air and oxygen intakes (6mm Ø).

The pressure-controlled air/oxygen mixture is admitted into the inspiratory branch via the electrovalve V1, which is also a common industrial component[1]. The valve is commanded by the electronic circuit described below. Downstream from V1 are installed the safety valve V3 (of the same mechanical design as V4, but spring-loaded instead of gravity-loaded), the mechanical manometer M1, the oximeter M2 and the connection to the electronic manometer that is part of the alarm system (described below).

Exhaled air is accepted from the expiratory branch through the electrovalve V2 (similar to V1), also electronically commanded, and rejected to the exterior via the positive end-expiratory pressure (PEEP) valve V4.

The PEEP valve is meant to provide a permanent backpressure in the patient lungs. Such valves are commercially available as consumables, but to reduce dependencies we designed (Figure 3S) a gravity check valve that provides fixed pressures of 0, 5, 10, 15 or 20cmH_2O via interchangeable calibrated lifts. These steps were defined based on the requirement in the "MHRA specifications"[1] that the adjustment of these pressures should have a maximum granularity of 5cmH_2O, indicating that smaller steps are clinically not meaningful.

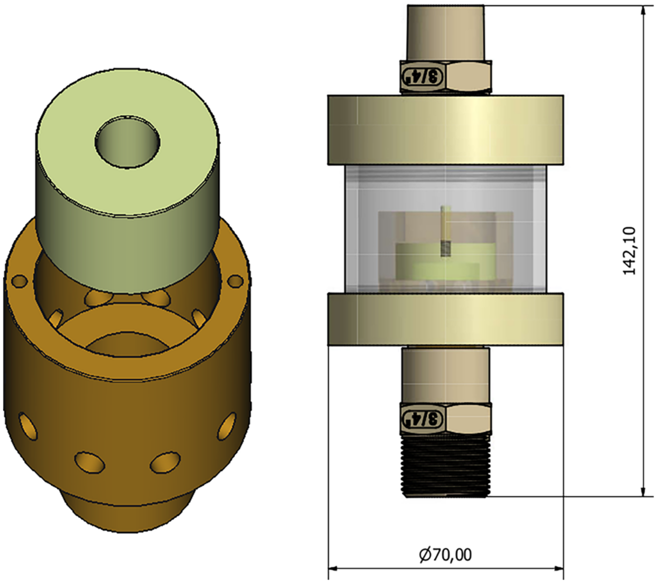

Figure 3S - 3D model of the sterilizable gravity-loaded PEEP valve.

Left panel: the valve siege and the weight-calibrated lift. Material: brass. Right panel: the gravity valve is kept in an easily removable closure to prevent contamination of the ventilator by the exhaled air. Materials: acrylic and POM plastics.

The valve is kept in a removable closure to prevent internal contamination of the ventilator by the exhaled air. The full expiratory branch (V2, V4 and tubing) can be easily removed and disassembled for sterilization (by immersion in alcohol, not steam) when switching patients. The coil of V2 can be removed from the valve body for this purpose.

To measure the tidal volume (V_T), an important parameter in patient care, a manual spirometer can be applied to the exhaust outlet (Figure 2S).

The ensemble is enclosed in an industrial-grade water-tight box, for protection and easy external cleaning in case of biological contamination.

Electronics

The timing of the electrovalves, defining the respiratory rate and the I/E ratio (duty cycle), is assured by the circuit schematically represented in figure 4S. In line with the design objective of simplicity and maintainability, the circuit uses only very common components that should be widely available worldwide.

It is composed by a symmetric almost-triangular wave generator based on a 555 timer that provides fixed respiratory rates of 12, 15, 18, 21 or 25 breaths per minute (bpm) selectable by a rotary switch. Permanent inspiration (CPAP mode) and expiration (OFF) positions are also foreseen. The triangular wave is then fed to a comparator that generates a rectangular wave with I/E of 1/2 or 1/3, selectable by a switch. More/other values of all parameters can be easily implemented by changing the value of the relevant resistors.

The rectangular wave then controls a MOSFET output stage that commands the electrovalves (easily accommodating any reasonable model), while also commanding a yellow visual indicator of the inspiratory cycle.

The user control panel can be seen in figure 2S and easily correlated with the schematics.

Owing to the difficulty in obtaining accurate capacitor values, accurate respiratory rates must be defined experimentally by adjusting the values of C10, C11 and C12 for one of the standard rates. The relative accuracy of the respiratory rates is then assured by the accuracy of the resistors.

A simple assist-control mode (selectable via the rotary switch) is implemented by the comparator on the upper right corner of figure 4S, which senses the output of the electronic manometer in the alarm circuit (see below). If the airway pressure descends below -2cmH_2O, indicative of an autonomous inspiration effort, a new cycle of respiration is initiated. The inspiration valve is kept open as long as the airway pressure is below this pressure, allowing some control by the patient of the duration of the inspiration phase and of the respiratory rate. In absence of such event, the circuit defaults to a 10bpm cycle.

Power can be provided by either a 12 VDC source or by an external transformer with 12 VAC secondary voltage. Both inputs can be connected simultaneously to provide uninterruptible power supply (UPS) functionality. The limits on these voltages and the current requirement are defined by the electrovalve coils (in the present prototype respectively 12 to 17 V and 1 A).

The apnea/power loss alarm circuit is represented in figure 5S. The output of the MPX5010 electronic manometer, connected to the inspiratory branch as shown in figure 1S, is fed to an oscillation detection circuit followed by a 15s timer. When the pressure wave is detected, a green visual indicator is lit, which appears to "respond" to the yellow inspiration indicator. If such detection is absent for more than 15s, indicative of an apnea situation (for instance, a disconnection of the patient tubing or a malfunction of the ventilator), a visual and auditory alarm is triggered. In case of power failure the alarm is also activated via Q1 and the supercapacitor C3 powers the alarm for at least 1 minute. The auditory alarm can be turned off, with visual indication.

All components are very common, except perhaps the electronic manometer and the supercapacitor.

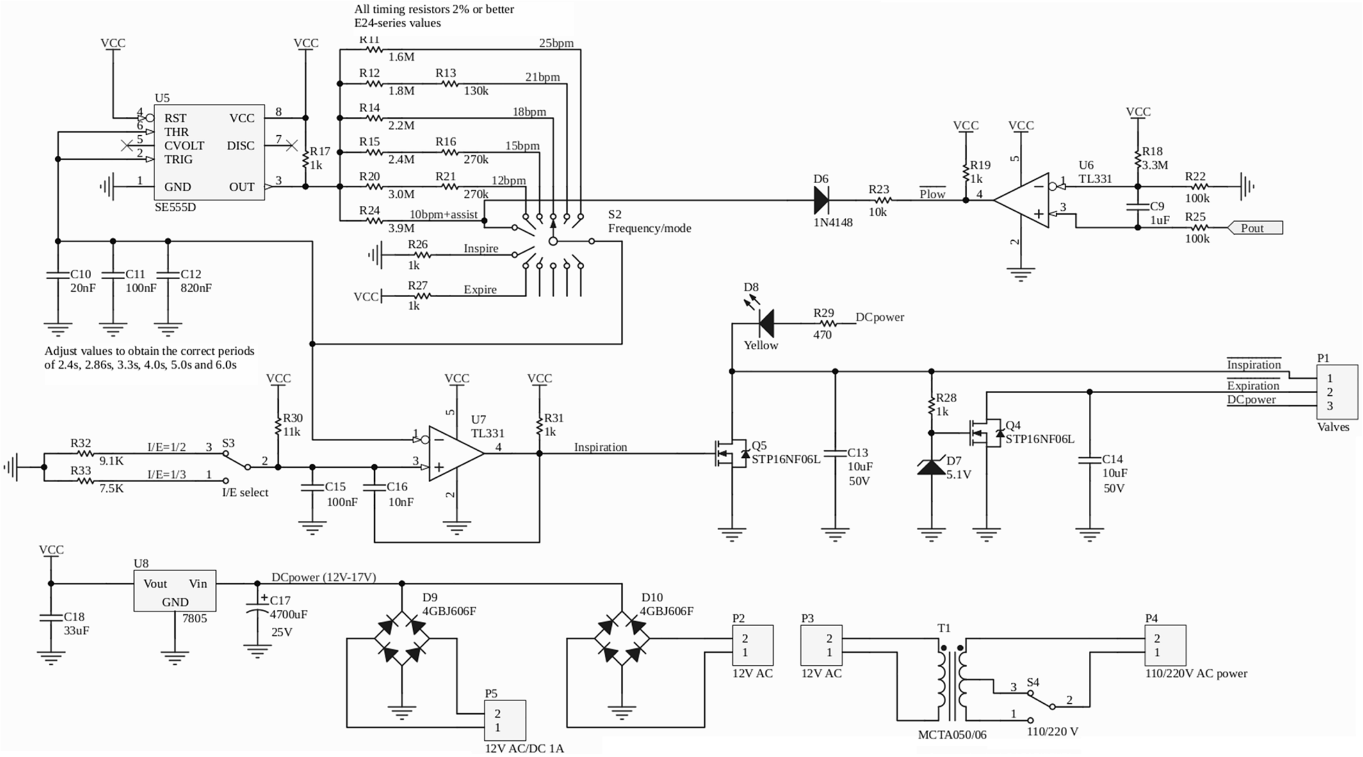

Figure 4S - Electrical schematic of the power supply and command of the ventilator electrovalves.

[1] BOEGGER SSPW21 mesh filter.
[2] Tecnogas, model LPZ7-37 (7kg/h - 37mbar). Available from: https://www.tecnogas.net/en/products/regolatore-bassa-pressione--19.
[3] Madas, model EVO/NC DN 20 (12 VDC, 8.5VA). Available from: https://www.madas.it/prodotti.php?ln=en&c=1&p=34.
[4] Medicines and Healthcare products Regulatory Agency. Specification for ventilators to be used in UK hospitals during the coronavirus (COVID-19) outbreak. Available from: https://www.gov.uk/government/publications/specification-for-ventilators-to-be-used-in-uk-hospitals-during-the-coronavirus-covid-19-outbreak.

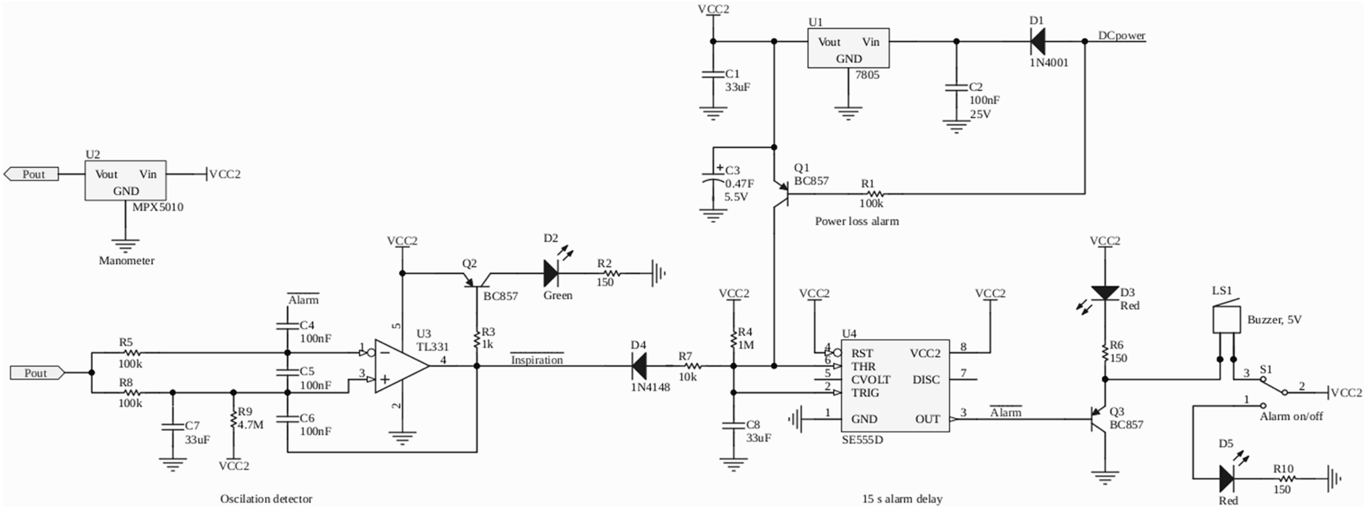

Figure 5S - Electrical schematic of the apnea/power loss alarm circuit.
